# Peripheral and Organ Perfusion’s Role in Prognosis of Disease Severity and Mortality in Severe COVID-19 Patients: Prospective Cohort Study

**DOI:** 10.3390/jcm13247520

**Published:** 2024-12-10

**Authors:** Mateusz Gutowski, Jakub Klimkiewicz, Bartosz Rustecki, Andrzej Michałowski, Tomasz Skalec, Arkadiusz Lubas

**Affiliations:** 1 Department of Anesthesiology and Intensive Care, Military Institute of Medicine-National Research Institute, 04-141 Warsaw, Poland; jklimkiewicz@wim.mil.pl (J.K.); brustecki@wim.mil.pl (B.R.); amichalowski@wim.mil.pl (A.M.); tskalec@wim.mil.pl (T.S.); 2 Department of Internal Diseases, Nephrology and Dialysis, Military Institute of Medicine-National Research Institute, 04-141 Warsaw, Poland

**Keywords:** sepsis, microcirculation, dynamic tissue perfusion measurement, perfusion, renal cortical perfusion, finger infrared thermography, capillary refill time

## Abstract

Severe COVID-19 is associated with a generalized inflammatory response leading to peripheral and organ perfusion disorders. **Objectives**: This study aimed to evaluate the usefulness of peripheral and organ perfusion assessments in the prediction of prognosis and mortality in patients with severe COVID-19. **Patients and Methods**: In the first 48 h of hospitalization, peripheral perfusion (saturation, Finger Infrared Thermography—FIT; Capillary Refill Time—CRT), and the color Doppler renal cortex perfusion (RCP) were estimated in a group of 102 severe COVID-19 patients. **Results**: In total, 40 patients experienced deterioration and required intensification of oxygen treatment, and 24 finally died. In comparison with a stable course of the disease, patients with deterioration had initially higher WBC, CRP, AST, LDH, and CRT, but a lower oxygenation ratio and RCP. Deceased patients were older, had higher CRP, LDH, and CRT, but lower hemoglobin, oxygenation ratio, and RCP compared to survivors. In the multivariable regression analysis from perfusion parameters, only RCP and CRT were independently linked with deterioration (OR 0.002, *p* < 0.001; OR 1.825, *p* = 0.003, respectively) and death (OR 0.001, *p* = 0.004; OR 1.910, *p* = 0.003, respectively). **Conclusions**: Initial assessment of peripheral and organ perfusion can be helpful in identifying hospitalized severe COVID-19 patients with a higher risk of deterioration and death. Capillary Refill Time and Renal Cortical Perfusion were prognostic markers of deterioration or death. On the other hand, Finger Infrared Thermography and saturation were not statistically significant in predicting patient outcome. An RCP cut-off value below 0.127 and 0.112 [cm/s] and a Capillary Refill Time longer than 3.25 and 4.25 [s] indicate the risk of deterioration or death, respectively.

## 1. Introduction

Patients with COVID-19 infection can have a wide range of symptoms, from asymptomatic to critical illness with multiple organ failure, respiratory failure, or septic shock [1]. In severe cases, COVID-19 can induce septic shock with an exaggerated inflammatory response and thrombotic disorders.

For the proper functioning of organs, their adequate perfusion and oxygen supply are fundamentally important [2,3]. Under physiological conditions, metabolic demand at the microcirculation level forces changes in the macrocirculation by influencing blood pressure or cardiac output, maintaining the body in homeostasis. One of the pathophysiological pathways of sepsis is a consequence of endothelial damage and glycocalyx disruption, increased vascular permeability, and intravascular coagulation [4,5].

This leads to profound tissue perfusion disorders. Many studies identify microcirculation disorders as the leading cause of hemodynamic insufficiency in septic shock [6]. Likewise, SARS-CoV2 leads to systemic changes mainly due to endothelial damage, which is particularly visible in the most severe cases [7].

In some patients, microcirculation disorders persist despite the normalization of macrocirculatory parameters [3]. It is worth noting that the disturbances mentioned above are associated with increased mortality in these patients. This argument stands in favor of studying the microcirculation of patients as a source of key information.

In cases with severe COVID-19, abnormal coagulation and thrombotic consequences are thought to be the main signs of endothelial dysfunction. For example, specimens from COVID-19 patients showed significant levels of circulating coagulant markers and thrombotic vessel occlusions, indicating the activation of coagulation after SARS-CoV-2 infection [8,9].

These changes in microcirculatory disturbances are generalized and affect all organs: lugs, brain, heart, gastrointestinal tract, kidneys, and liver [10,11,12]. As changes in peripheral perfusion seem to be of critical importance, strict monitoring of its variability should bring substantial information about the disease’s advancement.

Capillary Refill Time (CRT) is a quick, accessible test for peripheral perfusion assessment. It measures the amount of time that passes between the release of pressure from the fingernail and the restoration of normal color. The rate at which the capillary bed refills following compression determines the skin’s color return [13].

Scientific evidence demonstrates the value of CRT for patients experiencing shock, where substantial disruptions in peripheral flow are caused by an imbalance between vasodilators, vasoconstrictors, and endothelial cells. Numerous studies attest to the fact that determining peripheral perfusion during a physical examination using palpation and CRT aids the identification of individuals who are at a high risk of shock [14]. Signs of compromised peripheral perfusion detected using CRT are early markers of inadequate organ perfusion among patients in shock [15,16].

Finger infrared thermography (FIT) and pulse oximetry are other approved methods that could be used for peripheral perfusion assessment. The benefits of these measurements are that they are noninvasive, simple to use, inexpensive, and produce data that are objective [17,18].

Dynamic Tissue Perfusion Measurement (DTPM), a noninvasive technique based on Color Doppler ultrasound, was developed and effectively applied to evaluate renal cortex perfusion (RCP) as an equivalent of kidney blood flow [19,20]. This is an easily accessible, noninvasive test that has demonstrated its effectiveness in a range of clinical situations [21,22,23,24].

Risk factors for deterioration and mortality in COVID-19 patients have been extensively investigated across various dimensions. However, from an immunological perspective, the situation remains complex and not fully understood. An intense immunological response plays a significant role in the pathophysiology of COVID-19 [25]. Distinct cytokine profiles have been observed in association with disease severity [26]. Nevertheless, a meta-analysis conducted by Leisman et al. compared immunological responses across various conditions, including acute respiratory distress syndrome (ARDS) related and unrelated to COVID-19, sepsis, and cytokine release syndrome induced by chimeric antigen receptor T-cell. Their findings indicate that the immune response in COVID-19 is not particularly elevated compared to other diseases [27].

Despite this, promising studies continue to highlight the potential efficacy of treatments targeting the reduction in interleukin-6 (IL-6) concentrations [28].

From a clinical standpoint, pneumomediastinum has been shown to nearly double the fatality rate in COVID-19 patients. This phenomenon is likely linked to fibrous-hyaline degeneration of the tracheal rings, potentially driven by aberrant expression of the Wnt5a and Sonic Hedgehog signaling pathways [29].

Other factors may also influence the severity of COVID-19, including comorbidities, dietary habits, and physical activity [30,31,32].

The present study aimed to evaluate the usefulness of peripheral and organ perfusion assessments in the prediction of prognosis and mortality in patients infected with SARS-COV2.

This study aims to comprehensively explore the relationships between peripheral and organ perfusion and sepsis, with a specific focus on the context of COVID-19.

## 2. Materials and Methods

During the COVID-19 pandemic in 2021–2022, a prospective cohort study was conducted at the Temporary Hospital of the Military Institute of Medicine in Warsaw, Poland.

Only patients with a positive COVID-19 result, based on RT-PCR Test (GeneFinder COVID-19 Plus RealAmp Kit, OSANG Healthcare Co., LTD, Anyang, Republic of Korea), and with severe pneumonia requiring oxygen therapy were included. This study was conducted in accordance with the Declaration of Helsinki and was approved by the Bioethics Committee on 19 May 2021 (No. 22/WIM/2021). The inclusion criteria were intentionally broad, allowing for all patients under the age of 70 who consented to participate in the study to be eligible. The only criterion for exclusion was the lack of consent or its withdrawal.

Patients’ baseline measures were taken during the first two days of hospitalization. Information about medical history was taken from medical records. Measures included the following: physical examination, Capillary Refill Time (CRT), oxygen saturation measurement (pulse oximetry), a hand image taken with a thermal imaging camera (FIT—Finger Infrared Thermography), and Doppler ultrasound of the kidney using the dynamic tissue perfusion measurement (DTPM). The computer analysis of DTPM data was conducted after all patient data had been collected. Consequently, the attending physician was blinded to the results during the study. A routine CT scan of the lungs was performed in the emergency department among all included patients prior to admission.

### 2.1. Oxygenation Ratio—PaO_2_/FiO_2_

The oxygenation ratio was defined as the partial pressure of oxygen in arterial blood (PaO_2,_ mmHg) divided by the concentration of oxygen in the breathing mixture (FiO_2_).

This index is commonly used to compare the severity of changes in ARDS [33].

The New Global Definition of ARDS criteria use this indicator to categorize the stages of ARDS—0: >300; 1-mild: 300 > x > 200; 2-moderate: 200 > x > 100; 3-severe: >100.

### 2.2. Capillary Refill Time (CRT)

Firm pressure was applied to the ventral surface of the II and IV finger distal phalanx on both hands to assess CRT. The skin was pressed until it became blank, and then the pressure was maintained for five seconds. The amount of time before the skin’s original tone reappeared was then recorded. The outcome was determined by taking the mean of all the measures. [34].

### 2.3. Pulse Oximetry

Pulse oximetry was performed on each hand, and the result taken for analysis was the average value from the measurements. The Sanity Duo Control device (Albert Hohlkörper GmbH & Co., KG, Hemer, Germany) was used for this study.

### 2.4. Finger Infrared Thermography

**Figure 1:**
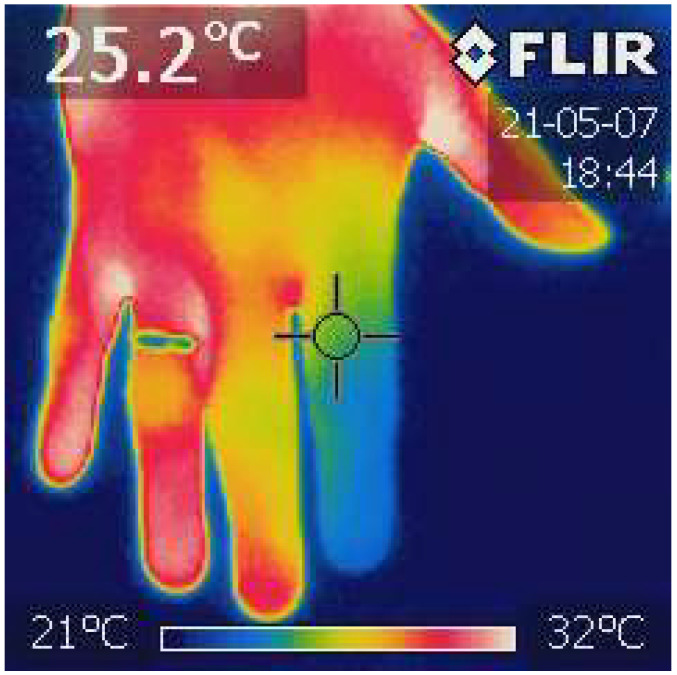
Finger Infrared Thermography (FIT). The photo was taken with the power of a thermal imaging camera (FLIR i7). The temperature of each distal phalanx was analyzed, and the average value was calculated. The ThermaBase application was used. Own materials.

The thermographic image of the hand was obtained with the use of the FLIR i7 thermal imaging camera (Teledyne FLIR LCC, Wilsonville, OR, USA). After calibrating the camera, a photo of each hand was taken from about 50 cm (Figure 1).

From the obtained images, the temperature of the distal phalanges was analyzed using the Image ThermaBase EU 3.0.0.59 software (Military Institute of Medicine-National Research Institute, Warsaw, Poland). The analyzed data were the average temperature value from all phalanges [35].

### 2.5. Ultrasound Examination

**Figure 2:**
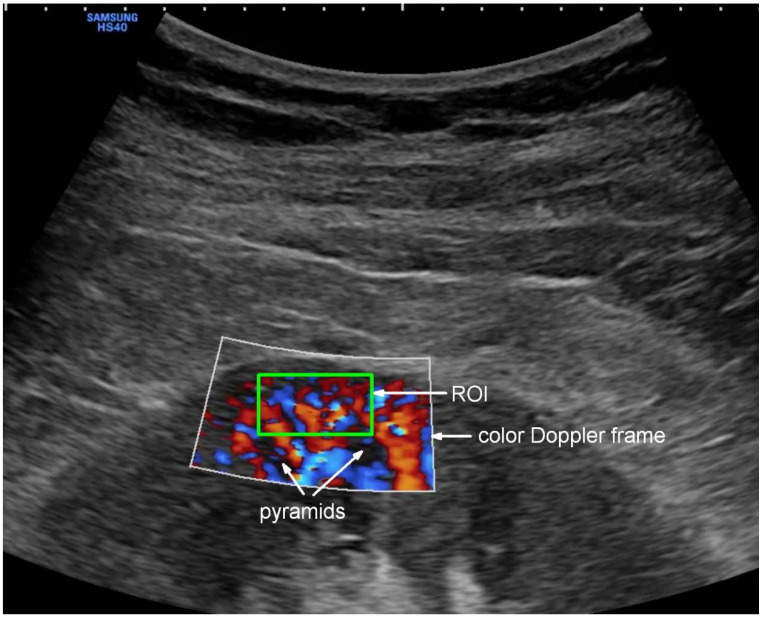
Color Doppler visualization of Renal Cortical Perfusion with selected ROI. The green box shows the location of the region of interest (ROI) for PixelFlux software (v. 18_03_11) flow quantification.

Doppler examination of the kidney was performed using the CA2-8AD convex probe (2–8 MHz) connected with Samsung HS40 (Suwon, Republic of Korea) equipment. The examined region of interest (ROI) was defined as an area of the kidney cortex between the outer edge of two pyramids and a kidney capsule containing interlobular vessels (Figure 2).

This image was obtained by acquiring the longitudinal cross-section of the right kidney. To keep the image stable, the patient was asked to stop breathing for several seconds. The gain of color Doppler was set constantly, unchanged so that the resulting images could be compared with each other. The flow velocity scale was set at 9.7 cm/s, subject to minor adjustments to optimally visualize the flow. Two to three videoclips in DICOM format were recorded, each lasting 3–5 complete heart cycles. The data collected in this way were used to calculate Renal Cortical Perfusion (RCP [cm/s])—expressing average arterial and venous flow intensity in the examined ROI using an external medical device (PixelFlux v. 18_03_11, Chameleon Software, Leipzig, Germany).

### 2.6. Statistical Analysis

Results are presented as means with standard deviations or medians with interquartile range (IQR) depending on fulfilling the normal distribution criteria. Otherwise, data are presented as numbers with percentage occurrence. The distribution normality was checked with the Shapiro–Wilk test, which has high power, especially for the lower groups. The *t*-test was used for independent and normally distributed variables; otherwise, the Mann–Whitney test was used for difference significance estimation. Univariable logistic regression analysis was performed to evaluate the prognostic properties of perfusion parameters. Then, variables significantly associated with outcomes were included in the backward multivariable logistic regression analysis model. Finally, to determine the optimal cut-off threshold predicting deterioration and mortality, ROC analysis was conducted using the Youden index and an equally high sensitivity and specificity option (EH) [36]. A double-sided *p*-value < 0.05 was considered statistically significant. All statistical analyses were performed with the use of Tibco Statistica v. 13.3 (TIBCO Software Inc., Greenwood Village, CO, USA).

## 3. Results

### 3.1. Descriptive Data: Demographics, Comorbidities, and ARDS Severity Based on the Oxygenation Ratio

The study included 102 patients (40F, 62M; age 58.5 ± 13.0), from which 40 (16F, 24M; age 60.0 ± 12.0) had deterioration and required intensification of oxygen treatment, and 24 (11F, 13M; age 61.9 ± 11.0) finally died during hospitalization. Comorbidities, course of disease, severity of COVID-19 infection, and mortality are presented in Table 1. Blood parameters and the results of the perfusion assessment in the considered groups are shown in Table 2.

In comparison to those with a stable course of disease, patients with deterioration had initially higher markers of inflammation (WBC, CRP), liver injury (AST) and organ hypoxia (LDH), but lower oxygenation ratio. Moreover, they had significantly lower results of peripheral and organ perfusion (longer CRT and lower RCP, respectively), suggesting that these parameters can have predictive properties for deterioration.

Considering deceased patients, they were older, had higher concentrations of CRP and LDH, more expressed lung involvement measured with oxygenation ratio, and prolonged CRT, but lower hemoglobin and renal cortex perfusion in comparison to survivors. Again, CRT expressing peripheral perfusion and RCP quantifying kidney cortex perfusion seemed to be useful in differentiating patients with good and bad prognosis.

### 3.2. Risk Factors for Clinical Deterioration and Mortality

Considering disease severity and outcome, no significant differences were observed in PLT, CK, saturation, kidney function, interdigital temperature, and blood pressure.

From initially estimated perfusion parameters, only CRT and RCP were significantly associated with the deterioration prognosis (Table 3).

The same variables (CRT and RCP) were independently correlated with deterioration in the model of the multivariable logistic regression analysis composed of all the considered perfusion parameters.

Similarly, only CRT and RCP were substantially associated with death prognosis in the univariable analysis (Table 3). Moreover, both CRT and RCP independently influenced the risk of death in the multivariable logistic regression analysis.

The higher the RCP, the lower the likelihood for deterioration (*p* = 0.002).

In overall and backward regression, RCP and CRT independently affected deterioration (*p* < 0.001; *p* = 0.003, respectively) and death (*p* = 0.004; *p* = 0.003, respectively).

The values of the proposed cut-off points are shown in Table 4 and Table 5.

**Figure 3:**
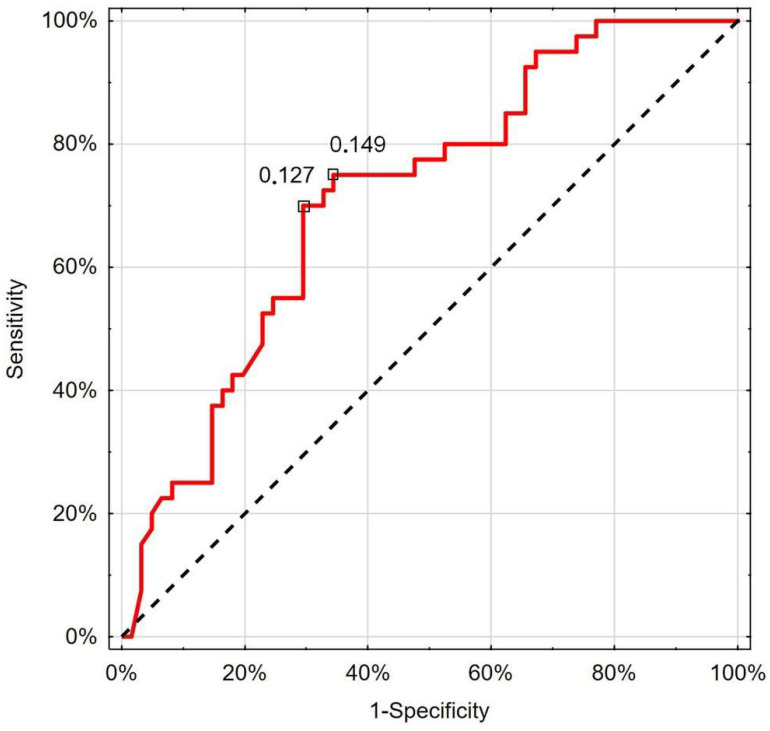
Receiver operating characteristic curve chart showing the discriminatory properties of RCP in diagnosing COVID-19 deterioration. Indicated values express thresholds investigated with EH (0.127 cm/s) and Youden index (0.149 cm/s) options.

The ROC analysis revealed that RCP with a nadir value of 0.127 cm/s had 70.0% sensitivity and 70.5% specificity (AUC 0.714; 95%CI 0.613–0.814; *p* < 0.001) in differentiating patients with deterioration (Figure 3).

**Figure 4:**
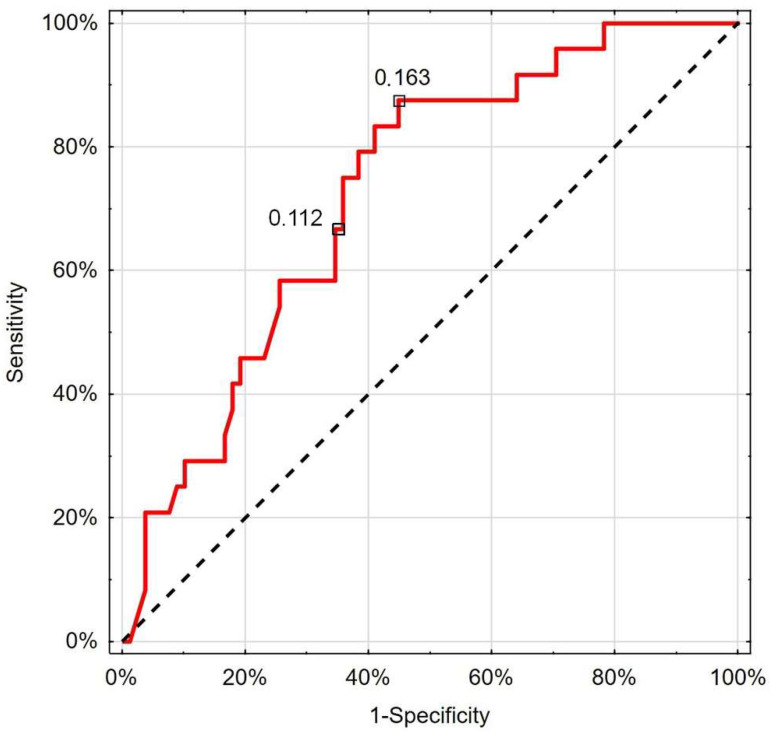
Receiver operating characteristic curve chart showing the discriminatory properties of RCP in diagnosing COVID-19 mortality. Indicated values express thresholds investigated with EH (0.112 cm/s) and Youden index (0.163 cm/s) options.

The performed ROC analysis showed that RCP below the cut-off value of 0.112 cm/s (66.7% sensitivity; 65.4% specificity) could indicate patients with worse survival prognosis (AUC 0.725; 95%CI 0.619–0.832; *p* < 0.001) (Figure 4).

**Figure 5:**
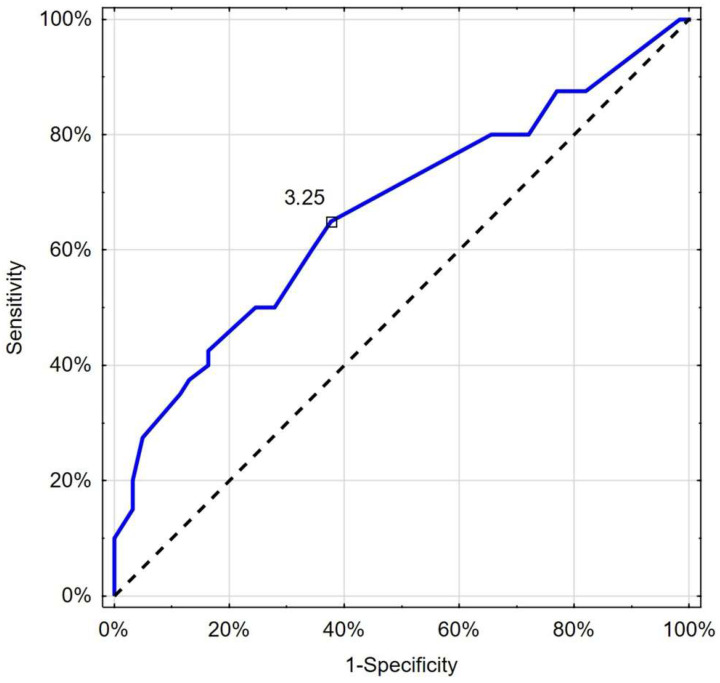
Receiver operating characteristic curve chart showing the discriminatory properties of CRT in diagnosing COVID-19 deterioration. Indicated values (3.25 s) express thresholds investigated with EH and Youden index options.

Moreover, in this analysis, CRT exceeding the cut-off value of 3.25 s was helpful in identifying patients with deterioration with 65.0% sensitivity and 62.3% specificity (AUC 0.667; 95%CI 0.555–0.778; *p* = 0.003) (Figure 5). There was no significant difference between the predictive properties of RCP and CRT in the case of deterioration (*p* = 0.338) or death (*p* = 0.534).

**Figure 6:**
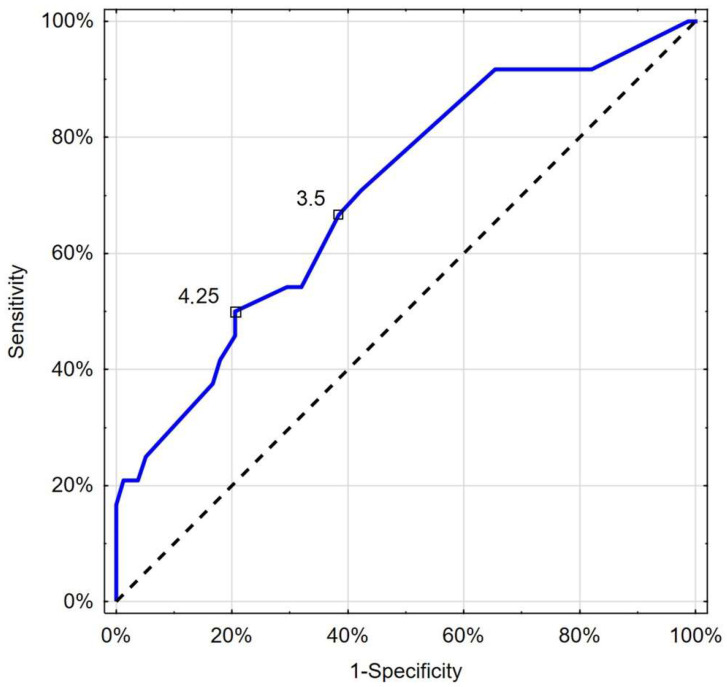
Receiver operating characteristic curve chart showing the discriminatory properties of CRT in diagnosing COVID-19 mortality. Indicated values express thresholds investigated with EH (3.5 s) and Youden index (4.25 s) options.

In addition, CRT over a nadir value of 3.5 s identified deceased patients with a comparable sensitivity of 66.7% and specificity of 61.5% (AUC 0.702; 95%CI: 0.581–0.823; *p* = 0.001) (Figure 6).

Phalangeal temperature measured using a thermal imaging camera was found to be statistically insignificant in predicting deterioration or death.

In a univariable logistic regression analysis, the relationship of saturation with deterioration was of a significant level (Table 3), and in the ROC analysis, saturation <94% appeared to be a predictor of deterioration (65% sensitivity and 52.5% specificity; AUC 0.618; *p* = 0.04). However, saturation was not independently associated with deterioration in the multivariable logistic regression analysis model (Table 3). The proposed saturation measured peripherally proved to be statistically insignificant in predicting death.

## 4. Discussion

In the present study, impaired organ and peripheral perfusion were significantly associated with deterioration and death in severe COVID-19 patients.

In multivariable backward regression analyses, RCP and CRT independently affected deterioration and death.

The Andromeda-Shock study opened a new field of research into the usefulness of CRT in fluid resuscitation and hemodynamic management of patients with sepsis, showing that it is non-inferior to the one based on lactate clearance [34]. In addition, secondary results indicated a lower severity of organ failure, as measured using the SOFA scale, during CRT-based resuscitation. Moreover, the post hoc analysis showed that patients with CRT-based resuscitation used less fluids, vasopressors, and had a lower risk of death than patients with lactate clearance [37]. With reference to Andromeda-Shock, Castro et al. conducted a randomized trial to evaluate CRT- or lactate-based fluid resuscitation in a population of 42 patients with septic shock [38].

Although the strategies were comparable, the advantage of CRT was the faster achievement of hemodynamic goals. In this study, the suggested CRT cut-off threshold was <3 s.

Our study showed increased mortality in patients with prolonged CRT. Such a relationship can be found in the literature, where it appears in the context of fluid resuscitation. Bakker et al. showed that abnormal CRT after fluid resuscitation was associated with 6 times greater mortality in comparison with patients with normal CRT [39]. Also noteworthy is a study by Ait-Oufella et al., in which the usefulness of CRT as a 14-day predictor of mortality was demonstrated, regardless of the catecholamine treatment [40].

CRT performed within 24 h of Intensive Care Unit (ICU) admission enables the identification of patients who will develop elevated blood lactate levels and severe organ failure [41]. Observational data have shown an association between prolonged CRT and increased mortality [41,42].

Our results show that RCP and CRT independently affected deterioration (*p* < 0.001; *p* = 0.003) and death (*p* = 0.004; *p* = 0.003), but peripheral temperature was not substantially helpful.

Similar conclusions can be drawn from the work of Brunauer et al., who used the pulsatility index of the kidney, liver, and spleen for organ perfusion. They showed correlations between peripheral flow (CRT, mottling score) and organ flow (visceral organs pulsatility index) in patients with septic shock [43]. However, DTPM can provide a more complete picture of renal perfusion because RI and PI can be measured in many interlobular vessels within the ROI, not just in a single segmental artery [20].

In a patient with shock, priority perfusion is diverted to the heart, lungs, and brain. Based on this, it is expected that there will be compensatory vasoconstriction in the peripheral vessels of the skin and visceral organs, leading to the redirection of blood flow [44]. Thus, evaluation of blood flow in those under-perfused organs can bring important information about the severity and advancement of the illness.

There are several reasons for choosing the kidney as the model for organ perfusion assessment.

About 1200 mL/min (20%) of cardiac output is made up of renal blood flow. Of this, about 80–90% flows through the renal cortex, where glomerular filtration occurs [45]. However, despite such a rich flow, the kidneys are still relatively susceptible to ischemic damage. Ischemic injury is one of the mechanisms of acute kidney injury (AKI), which is one of the most severe complications in critically ill patients—it is associated with increased mortality, risk of developing chronic kidney disease, and end-stage kidney disease [46].

To sum up, the proper flow through the kidney is characterized by a high-volume flow with low resistance, and its disruption can induce AKI. Thus, it should be routinely tested.

Secondly, the anatomy of the renal cortex vessels is advantageous for measurement because the arteries run perpendicular to the ultrasound probe, so there is less risk of measurement error [20].

Poorer peripheral flow has been shown to correlate with decreased creatinine clearance [20]. In addition, relatively simple anatomical access to the US kidney makes for an optimal target for organ perfusion testing.

There are a few noninvasive methods quantitatively assessing renal blood flow [47]. The most used is conventional duplex or triplex Doppler of segmental intrarenal arteries with the estimation of Pulsatility and Resistance Indices (PI, RI), contrast-enhanced ultrasonography, or DTPM [20].

Widely accessible RI and PI measurements, although very useful, have some significant limitations. The most important is a single point and single vessel measurement, with the systolic and end-diastolic velocities assessment, without vascular diameter. Thus, it represents only some flow properties and has to be repeated in 2–3 vessels and then averaged.

However, RI and PI do not directly reflect the flow through the cortex of the kidney, which can lead to erroneous conclusions and further improper clinical decisions [20,22].

It should be noted, however, that changes in the microcirculation flow resulting from increased flow heterogeneity and increasing capillary insufficiency characterize important mechanisms of sepsis-associated AKI [48,49]. This leads to ischemia of the nephron, causing loss of function and damage to the epithelial cells of the renal tubules [48,50].

An alternative method of assessing perfusion is contrast-enhanced sonography. In this method, perfusion intensity is evaluated indirectly from contrast enhancer influx curves (steepness of influx and level of saturation). This enables the measurement of perfusion intensity in all vessels of a larger ROI. However, this method does not allow for investigation of the flow velocity or heartbeat dynamics because the calculations are based on contrast saturation curves. In addition, the method is an invasive burden, with measurement errors resulting from the use of contrast and generating additional costs [20].

Dynamic Tissue Perfusion Measurement (DTPM) is a promising and adequate ultrasound method that is still not widely used in assessing different parameters of Renal Cortical Perfusion in the given Region of Interest (ROI) [20,22]. Based on perfusion visualized in Color Doppler, an external software can calculate cortical RI, PI, vascular area, flow velocity, and intensity, which is equivalent to Renal Cortical Perfusion (RCP).

Although conventionally estimated, Pulsatility and Resistive Indexes are significantly correlated with the severity of chronic kidney disease, DTPM may be more useful in the dynamic process of sepsis [51]. Nevertheless, the assessment of renal perfusion in DTPM has not been already studied in a septic state. However, a Duplex Doppler study conducted by researchers led by Brunaer et al., showed a significant relationship between the pulsatility index of visceral organs (kidney, liver, spleen, and intestine), CRT, and mottling score, but not peripheral temperature [43]. Moreover, in severe COVID-19 lung involvement, we have shown that RCP impairment was the only independent perfusion factor associated with the stage of ARDS [52]. In addition, the usefulness of organ perfusion assessment in the DTPM method in different clinical situations has been introduced in the cardiorenal axis, in which significant associations between RCP and the systolic and diastolic effects of the left ventricle and cardiac output have been demonstrated [53,54]. In other studies, RCP appears to be a sensitive marker of kidney function in a hypothyroid state and isotope-related kidney injury [55,56].

In our study, the temperature of the phalanx (FIT), estimated using a thermal imaging camera, did not indicate a risk of deterioration or death. In addition, studies regarding peripheral temperature and prognosis are inconsistent. Measuring peripheral temperature was helpful in Joly and Weil’s work, which examined the cold toe as a parameter of the severity of circulatory shock [57]. Bourcier et al. measured the toe-to-room temperature gradient and showed that it is an independent risk factor for mortality in critically ill patients with sepsis and septic shock [58]. Houwink et al. studied delta-T, the difference between central and forefoot temperatures, as an independent factor in ICU mortality in septic patients [59]. On the other hand, Brunaer et al. showed that in septic patients, organ perfusion and CRT are preserved, but not peripheral temperature [43].

In this context, it is worth quoting the study by Boerma et al., who found no correlation between the sublingual microcirculatory alteration and the central-to-toe temperature difference in septic and septic shock patients [60]. The authors emphasize the roles of differential flow between microcirculation and systemic flow caused by sepsis.

We showed that for mortality prediction, CRT > 3.5 s has a sensitivity of 66.7% and specificity of 61.5% (AUC 0.702; 95%CI: 0.581–0.823; *p* = 0.001). In the study by Hiemstra et al., prolonged CRT > 4.5 s was shown to be one of the clinical indicators of reduced Cardiac Index [61]. Lara et al. conducted a prospective study on 95 septic patients admitted to the emergency department. Patients underwent fluid resuscitation. In patients with hyperlactatemia and persistent prolonged CRT, worse clinical outcomes and higher mortality were observed [62].

In a study of 132 children with sepsis, Fernández-Sarmiento et al. examined CRT and assessed microcirculation using videomicroscopy. A link between prolonged CRT and changes in microcirculation has been demonstrated [63]. Glycocalyx damage and reduced capillary recruitment were observed in these patients. However, no statistically significant relationship with mortality was found.

In our study, the performed ROC analysis indicates that the threshold value for RCP < 0.127 cm/s had 70.0% sensitivity and 70.5% specificity (AUC 0.714; 95%CI 0.613–0.814; *p* < 0.001) in differentiating patients with the deterioration. Moreover, this cut-off value was even lower.

(RCP < 0.112 cm/s; 66.7% sensitivity; 65.4% specificity) for proper indication of patients with worse survival prognosis (AUC 0.725; 95%CI 0.619–0.832; *p* < 0.001).

Different cut-off points for CRT and RCP were proposed in the setting of deterioration or death occurrence. This can be summarized by the conclusion that the more impaired the CRT or RCP, the correspondingly higher the risk of deterioration or death.

As we mentioned above, renal cortex DTPM has not been studied in septic patients, so we do not have any literature directly correlating this topic, but it is worth looking at the researchers who have explored AKI in septic patients using contrast-enhanced ultrasound.

Watchorn et al. demonstrated, in COVID-19 patients with AKI, that renal perfusion, both macro- and micro-, is reduced, regardless of cardiac output or right ventricular function [64].

Watchorn et al. continued their study, but this time on a population of critically ill patients with sepsis and AKI, presenting analogous findings, where AKI was associated with reduced renal perfusion with preserved cardiac output and renal macrocirculation [65]. An interesting observation was made by A. Harrois et al. investigating the renal perfusion of patients in septic shock. They found that in these patients, when shock is present, the flow through the renal cortex can be normal, decreased, or increased, with preserved cardiac output [23]. However, in the subgroup of patients who additionally had AKI, the flow through the renal cortex was lower.

Although the presented results are promising, our study has some limitations.

CRT and ultrasound examinations are operator-dependent and were performed by the same researcher in our study.

Thus, interobserver variability of achieved data cannot be tested. It should be emphasized that we investigated infectious patients, which required us to wear personal protective clothing, which made it difficult to perform the exam.

In addition, this prevents us from repeating measurements and to assess intraobserver differences.

Moreover, it is worth noting the heterogeneity of the course of COVID-19. Although all our patients required oxygen therapy, there is a significant discrepancy between the severity of the disease in individual patients.

The strength of this study is an attempt to evaluate perfusion holistically using modern techniques. We employed a range of diagnostic techniques, including Capillary Refill Time (CRT), oxygen saturation, Fingertip Infrared Thermography, and Ultrasound examination, followed by post hoc computational analysis. While this study was conducted exclusively with COVID-19 patients, the methodology is readily adaptable to other clinical settings.

Although the presented results are promising, our study has several limitations. Both CRT and ultrasound examinations are inherently operator-dependent, and in this study, all measurements were performed by a single researcher.

Ultrasound examination is subject to various sources of error, including technical, operator-dependent, and patient-related factors.

Among these, patient-related factors warrant further discussion. First, obesity presented a noteworthy challenge. A thicker layer of adipose tissue significantly reduced the accuracy of the examination. Second, motion artifacts further compromised the reliability of certain measurements. It is also important to consider the heterogeneity in the clinical course of COVID-19 among our patients. While all participants required oxygen therapy, the severity of their conditions varied widely—from those requiring only a nasal cannula to those needing high-flow nasal oxygen (HFNO) or mechanical ventilation. This variability affected the patients’ ability to maintain breath-hold during the examination. To address this issue, multiple ultrasound assessments were performed for each patient, and the most reliable results were selected for analysis.

Operator-dependent limitations also played a role. These include the inherent variability of examination techniques and the physical constraints imposed by personal protective clothing (PPE). As the study involved infectious patients, PPE was mandatory, which further hindered the precision of the procedures and limited the ability to repeat measurements. Consequently, interobserver variability could not be assessed, and intraobserver differences were also challenging to evaluate.

## 5. Conclusions

Decreased peripheral and organ perfusion is associated with a higher risk of deterioration and death in hospitalized patients with severe COVID-19. Thus, initial assessment of peripheral and organ perfusion can be helpful in identifying those patients with worse prognosis. Capillary Refill Time and color Doppler Renal Cortical Perfusion are easily accessible and adequate parameters for evaluating peripheral and organ perfusion, which are independently associated with the risk of deterioration and death of severe COVID-19 patients. On the other hand, Finger Infrared Thermography and Finger Oxygen Saturation appear to have limited, nonsignificant usefulness for outcome prediction in this patient population.

## Figures and Tables

**Table 1:** Descriptive data, demography, diseases, and ARDS severity based on Oxygenation Ratio. Comparison of blood, clinical, and perfusion results among the investigated population.

Variable		n Median (Mean)	% IQR (±SD)
Gender	Male	62	60.8
	Female	40	39.2
Ward Type	Intensive Care Unit	34	33.3
	High-Dependency Unit	68	66.7
Deterioration		40	39.2
	HFNO/NIV	17	16.7
	Invasive Mechanical Ventilation	33	32.4
Death		24	23.5
Comorbidity	Malignancy	11	10.8
	Obesity	14	13.7
	Chronic Kidney Disease	5	4.9
	Coronary Artery Disease	7	6.9
	Heart Failure	6	5.9
	Myocardial infarction	2	2.0
	Atrial fibrillation	7	6.9
	Hypertension	32	31.4
	Diabetes	19	18.6
	Asthma	7	6.9
	Chronic Obstructive Pulmonary Disease	5	4.9
	Smoker	3	2.9
ARDS	No ARDS (PaO_2_/FiO_2_ > 300)	31	30.4
	MILD (PaO_2_/FiO_2_ 300 to 200)	22	21.6
	MODERATE (PaO_2_/FiO_2_ 200 to 100)	25	24.5
	SEVERE (PaO_2_/FiO_2_ < 100)	24	23.5
Age [years]		61	21
WBC [1 × 10^9^/L]		7.62	6.13
Hgb [g/dL]		13.6	3.2
PLT [1 × 10^9^/L]		186	111
CRP [mg/dL]		7.4	10.9
Creatinine [mg/dL]		0.9	0.4
Urea [mg/dL]		36	27
AST [U/L]		45	35
ALT [U/L]		35	25.5
CK [U/L]		168	386
LDH [U/L]		394	251
SBP [mmHg]		129	21.5
DBP [mmHg]		(79)	(12)
Oxygenation Ratio		211.5	221
Saturation [%]		95	5
CRT [s]		3	1.75
FIT [°C]		32.35	4
RCP [cm/s]		0.148	0.221

HFNO—High-Flow Nasal Oxygen; NIV—Noninvasive Ventilation; FiO_2_—Fraction of Inspired Oxygen; PaO_2_—Pressure of Oxygen; Oxygenation Ratio—PaO_2_/FiO_2_. WBC—White Blood Cells; Hgb—Hemoglobin; PLT—Platelets; CRP—C-Reactive Protein; AST—Aspartate Aminotransferase; ALT—Alanine Transaminase; CK—Creatine Kinase; LDH—Lactate Dehydrogenase; SBP—Systolic Blood Pressure; DBP—Diastolic Blood Pressure; CRT—Capillary Refill Time; FIT—Finger Infrared Thermography; RCP—Renal Cortical Perfusion.

**Table 2:** Comparison of blood, clinical, and perfusion results among patients with deterioration and those who were deceased.

	Non-Deterioration n= 62		Deterioration n = 40		*p*-Value	Survived n = 88		Deceased n = 24		*p*-Value
	Median (MEAN)	IQR (±SD)	Median (MEAN)	IQR (±SD)		Median (MEAN)	IQR (±SD)	Median (MEAN)	IQR (±SD)	
Age [years]	58.0	13.4	63.5	17.5	0.359	57.5	13.5	65.0	13.0	0.099
WBC [1 × 10^9^/L]	6.05	5.76	8.61	5.61	0.005	6.48	5.21	9.37	7.15	0.124
Hgb [g/dL]	13.6	3.5	(13.2)	(1.96)	0.704	13.9	3.3	12.9	2	0.044
PLT [1 × 10^9^/L]	177.5	123.0	191.0	85.5	0.550	186	121	185	94	0.940
CRP [mg/dL]	5.3	8.6	11.6	11.2	0.002	6.4	0.3	(11.6)	(7.6)	0.057
Creatinine [mg/dL]	0.8	0.3	1.0	0.6	0.195	0.9	0.4	0.95	0.07	0.829
Urea [mg/dL]	33.0	22.5	42.0	32.5	0.055	35	21	44.5	44.5	0.086
AST [U/L]	36.5	29.5	58	37	0.005	40	37	48.5	32.0	0.781
ALT [U/L]	31.5	25.0	38	24	0.176	33	26	39.5	22.0	0.564
CK [U/L]	140	355	243	475	0.482	182.0	447.5	166.0	345.0	0.466
LDH [U/L]	(350.8)	(147.0)	547.5	194	<0.001	367	214	(526.2)	(166.7)	0.002
SBP [mmHg]	129	27.5	(127)	(13)	0.438	130	25	127	11	0.866
DBP [mmHg]	(80)	(14)	(77)	(10)	0.242	(79)	(13)	(77)	(11)	0.469
Oxygenation Ratio	(272.8)	(98.4)	100	58	<0.001	269	212	92.5	53	<0.001
Saturation [%]	(94)	(3)	(93)	(3)	0.198	95	4	(93)	(3)	0.421
CRT [s]	3.0	1.25	(4.0)	(1.4)	0.005	3.0	1.5	(4.3)	(1.5)	0.003
FIT [°C]	31.8	3.9	33.0	4.9	0.350	31.9	4.0	33.2	5.2	0.806
RCP [cm/s]	0.187	0.281	0.061	0.136	<0.001	0.181	0.259	0.055	0.113	<0.001

**Table 3:** Results of univariable and multivariable logistic regression analysis of perfusion parameters and deterioration.

	Deterioration						Death					
Variable	Univariable Analysis			Multivariable Analysis			Univariable Analysis			Multivariable Analysis		
	OR	95%CI	*p*	OR	95%CI	*p*	OR	95%CI	*p*	OR	95%CI	*p*
Saturation [%]	0.863	0.743–1.003	0.055	-	-	-	0.888	0.752–1.049	0.161	-	-	-
CRT [s]	1.697	1.201–2.398	0.0027	1.825	1.227–2.714	0.003	1.814	1.244–2.644	0.002	1.910	1.249–2.921	0.003
FIT [°C]	1.000	0.884–1.132	0.997	-	-	-	0.971	0.845–1.116	0.677	-	-	-
RCP [cm/s]	0.002	0.000–0.080	0.0007	0.002	0.000–0.067	0.001	0.001	0.000–0.110	0.004	0.001	0.000–0.108	0.004

**Table 4:** Results of RCP ROC analysis for deterioration and mortality.

Prediction Target	RCP Cut-Off Value [cm/s]	Method	Sensitivity [%]	Specificity [%]
Deterioration	0.127	EH	70.0	70.5
	0.149	Youden	75.0	65.6
Mortality	0.112	EH	66.7	65.4
	0.163	Youden	87.5	55.1

EH—cut-off point whose sensitivity and specificity are equally high.

**Table 5:** Results of CRT ROC analysis for deterioration and mortality.

Prediction Target	CRT Cut-Off Value [s]	Method	Sensitivity [%]	Specificity [%]
Deterioration	3.25	Youden, EH	65.0	62.3
Mortality	3.5	EH	66.7	61.5
	4.25	Youden	50	79.5

EH—cut-off point whose sensitivity and specificity are equally high.

## Data Availability

The dataset is maintained by the authors and available on request.
